# Neuropathological correlates of imaging in dementia with Lewy bodies

**DOI:** 10.1007/s00702-026-03237-6

**Published:** 2026-07-17

**Authors:** Lauren Walker, Johannes Attems, Paul Donaghy

**Affiliations:** 1 School of Health and Life Sciences, Middlesbrough Tower, Teesside University, Middlesbrough, TS1 3BB UK; 2 Translational and Clinical Research Institute and NIHR Newcastle Biomedical Research Centre, Newcastle University, Newcastle upon Tyne, UK; 3 Institute of Neuropathology and Neuromolecular Pathology, Medical University, Innsbruck, Austria

**Keywords:** Dementia with Lewy bodies, Neuroimaging, Neuropathology, Autoradiography

## Abstract

Dementia with Lewy bodies (DLB) is a common yet underdiagnosed neurodegenerative dementia, characterised neuropathologically by nigrostriatal dopaminergic degeneration and widespread α-synuclein aggregation. Clinical diagnosis remains challenging due to marked heterogeneity in presentation and substantial overlap with Alzheimer’s disease (AD), resulting in diagnostic delays and frequent misdiagnosis. The ability to identify underlying pathological processes ante-mortem is essential to improve diagnostic accuracy, refine prognosis, stratify patients for clinical trials, and ultimately enable personalised therapeutic approaches. Over recent decades, major advances have been made in the development of neuroimaging biomarkers that reflect key features in Lewy body disease pathology, many of which have informed diagnostic criteria. In addition to dopaminergic dysfunction and α-synuclein pathology, co-existing neuropathologies including amyloid-β, tau, neuroinflammation, and vascular changes are increasingly recognised as important modifiers of clinical phenotype and disease trajectory in DLB. This review summarises the current landscape of neuroimaging modalities in DLB and critically examines their relationships with confirmed neuropathological correlates. We discuss indicative biomarkers such as striatal dopamine imaging and ^123^I-metaiodobenzylguanidine myocardial scintigraphy, alongside supportive imaging markers derived from structural magnetic resonance imaging and Fluorodeoxyglucose Positron Emission Tomography (FDG-PET). Emerging PET approaches targeting α-synuclein, as well as established amyloid and tau-PET techniques for assessing co-pathologies, are reviewed in the context of post-mortem validation studies. Finally, we consider Translocator protein PET imaging as a marker of neuroinflammation and highlight key methodological limitations and knowledge gaps. Together, these neuroimaging–pathological correlations provide critical insights into disease mechanisms and outline future directions toward earlier, biologically informed diagnosis and precision medicine in DLB.

## Introduction

**Fig. 1:**
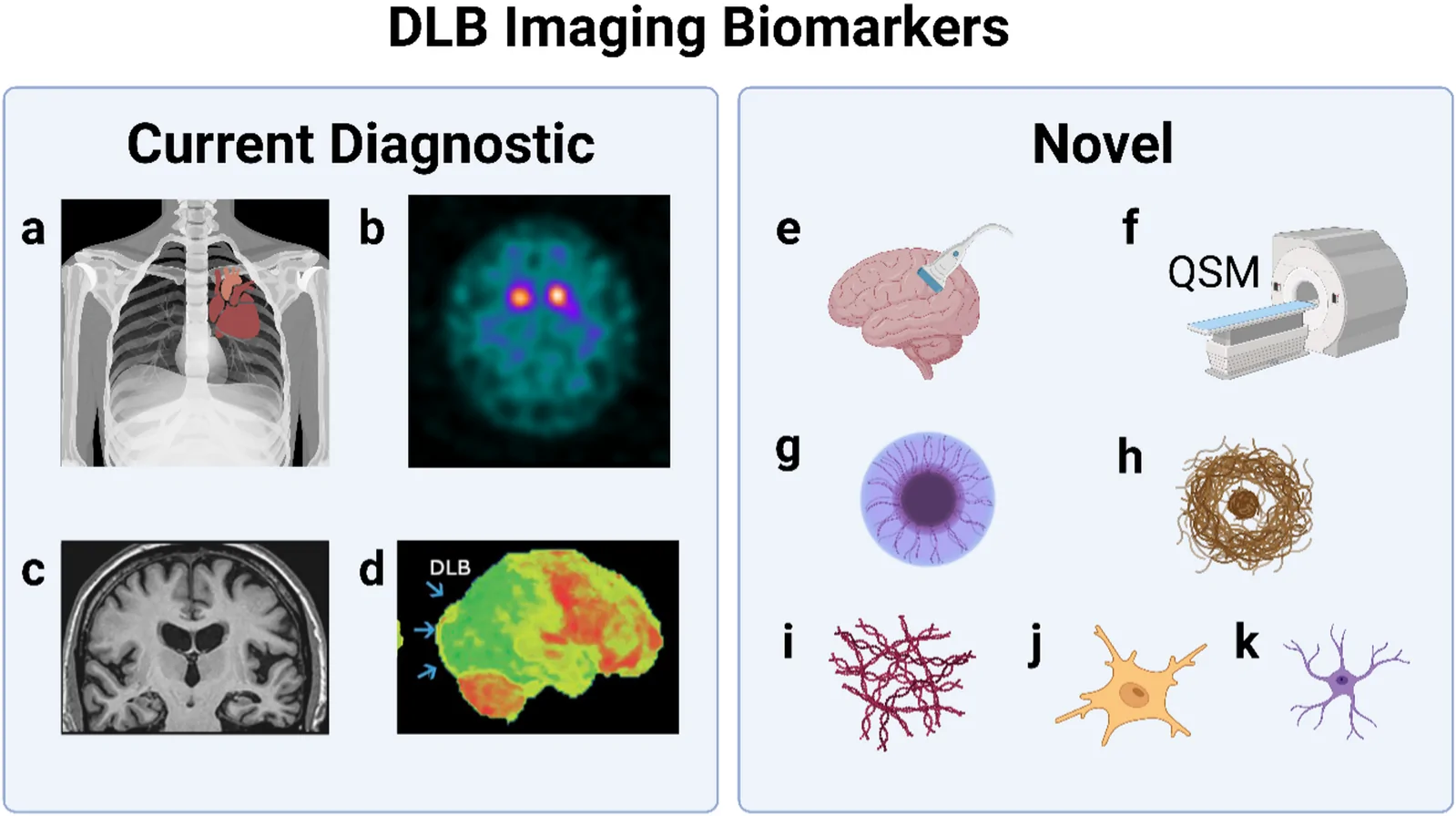
Schematic overview of current and emerging neuroimaging modalities in dementia with Lewy bodies (DLB). Clinically established indicative biomarkers include ^123^I-meta-iodobenzylguanidine (^123^I-MIBG) cardiac scintigraphy (**a**) and striatal dopamine transporter imaging (**b**). Supportive biomarkers routinely used in clinical assessment include structural magnetic resonance imaging (MRI) (**c**) and perfusion or metabolism imaging, e.g. fluorodeoxyglucose positron emission tomography (FDG-PET) or ^99m^Tc-hexamethylpropyleneamine oxime single-photon emission computed tomography (HMPAO SPECT) (**d**). In contrast novel structural and biophysical approaches in the UK include transcranial sonography (**e**) and quantitative susceptibility mapping (QSM) (**f**). Molecular imaging approaches include PET imaging targeting α-synuclein (**g**) amyloid-β (Aβ) (**h**), hyperphosphorylated tau (**i**), microglial activation (**j**), and astrocytic activity (**k**). Created with BioRender.com

The neuropathological correlates of dementia with Lewy bodies (DLB) have been known since the discovery of dopaminergic loss in the substantia nigra (SN) and subsequent degeneration of the nigrostriatal pathway. Furthermore, since the discovery that α-synuclein is the main component of Lewy bodies in idiopathic Parkinson’s disease (PD) (Spillantini et al. 1997) the research field has been striving to find a biomarker to accurately diagnose cases earlier so that patients will be able to benefit from disease modifying therapies as and when they become available. Over the years great strides have been made in developing neuroimaging biomarkers that accurately reflect the underlying disease mechanisms in patients with DLB, some of which have been incorporated into the diagnostic criteria (Outeiro et al. 2019). In addition to dopaminergic loss and α-synuclein aggregation, other neuropathological changes have been identified in DLB which can alter the clinical phenotype and disease trajectory (Outeiro et al. 2019). Promising neuroimaging techniques have the potential to identify these changes in vivo (Fig. 1). To provide accurate diagnosis, prognosis and give us the ability to provide personalised medicine, it is vital we are able to identify pathological changes ante-mortem. Therefore, this review will cover the current landscape of neuroimaging modalities and how they relate to the neuropathological correlates in DLB.

## Overlap of clinical symptoms between DLB and AD

The overlapping clinical features between DLB and Alzheimer’s disease (AD) make the diagnosis of DLB challenging and it is therefore often misdiagnosed. Neuropathological studies suggest that DLB accounts for 15–20% of dementia cases (Aarsland et al. 2001; Jellinger and Attems 2011). Most of these cases do not receive a clinical diagnosis, with diagnoses ranging from 2.9% to 5.4% in different centres within the UK, which may reflect differences in diagnostic practice (Kane et al. 2018). Furthermore it is reported in a study by Galvin and colleagues that 33% of patients visit a clinician six or more times before a diagnosis is given, with 20% of patients waiting for more than three years for a formal diagnosis (Galvin et al. 2010). Therefore, accurate antemortem prediction of underlying disease mechanisms will aid diagnosis, prognosis, help to stratify patients for clinical trials, and monitor efficacy and endpoints in clinical trials.

## Current diagnostic imaging biomarkers

### Indicative imaging biomarkers

#### Striatal dopaminergic imaging

Nigrostriatal dopaminergic loss can be assessed in DLB using positron emission tomography (PET) and single photon emission computed tomography (SPECT) with ligands such as.

^123^I-N-fluoropropyl-2b-carbomethoxy-3b-(4-iodophenyl) nortropane (FP-CIT) and can distinguish DLB from other neurodegenerative diseases such as AD (McKeith et al. 2007).

In a study of 55 patients (33 cases diagnosed as DLB and 22 diagnosed as AD) who underwent ^123^I-FP-CIT and consequently donated their brain at autopsy diagnosis, ^123^I-FP-CIT had a balanced diagnostic accuracy of 86% (sensitivity 80%, specificity 92%) against autopsy diagnosis compared with clinical diagnosis, which had an accuracy of 79% (sensitivity 87%, specificity 72%) (Thomas et al. 2016).

The pathological underpinnings of the imaging changes have been validated, demonstrating a relationship with neuronal loss and decreased cell counts. Colloby and colleagues investigated this in a study in 2012 (Colloby et al. 2012), where subjects underwent ante-mortem dopaminergic scanning and post-mortem assessment for dopaminergic cell loss and burden of neurodegenerative pathologies. In striatal regions, imaging uptake was associated with nigral dopaminergic neuronal density but not tau, amyloid β (Aβ), or α-synuclein pathology suggesting decreased in vivo uptake is not influenced by the presence of pathological lesions. This has been replicated in further post mortem analysis, where the correlation between striatal dopamine transporter SPECT and post mortem substantia nigra (SN) cell counts was very high (*r* = 0.96-98) (Kraemmer et al. 2014).

#### ^123^I-metaiodobenzylguanidine myocardial scintigraphy

^123^I-meta-iodobenzyl-guanidine (^123^I-MIBG) a physiologic analog of norepinephrine (Vallabhajosula and Nikolopoulou 2011) is a radiopharmaceutical to quantify postganglionic sympathetic cardiac innervation (Lurie et al. 1995). ^123^I-MIBG myocardial scintigraphy is a technique used as a diagnostic imaging test to differentiate Lewy body diseases (LBDs), including PD and DLB, from other similar diseases (McKeith et al. 2017). To validate this imaging biomarker against the gold standard of neuropathology Matsubara and colleagues compared ^123^I-MIBG myocardial scintigraphy findings with autopsy findings of 56 patients (30 with pathologically confirmed DLB and 26 controls) (Matsubara et al. 2022). In addition, the proportion of residual tyrosine hydroxylase (TH)-immunoreactive sympathetic fibres in the anterior wall of the left ventricle was investigated to assess the condition of the cardiac sympathetic nerves. The early heart-to-mediastinum (H/M) ratio had a sensitivity and specificity of 70% and 96%, whilst the delayed H/M ratio has sensitivity and specificity of 80% and 92% respectively. In addition, the proportion of residual TH-immunoreactive cardiac sympathetic fibres strongly correlated with the amount of cardiac ^123^I-MIBG uptake when assessed with early and delayed H/M ratio values. Whilst this study validates ^123^I-MIBG myocardial scintigraphy as a neuropathologically confirmed biomarker of cardiac innervation, to date no post-mortem validation study has been undertaken to assess the association with α-synuclein pathology in the brain, however some studies have suggested that serum and skin α-synuclein deposits are related to ^123^I-MIBG myocardial scintigraphy (Giannoccaro et al. 2020; Park et al. 2022).

### Supportive imaging biomarkers

### Structural magnetic resonance imaging

The preservation of the medial lobe as part of a signature pattern of atrophy on magnetic resonance imaging (MRI) is a feature that discriminates AD from DLB (Whitwell et al. 2007; Oppedal et al. 2019). To determine the neuropathological correlates of regional medial temporal lobe changes on MRI in patients with DLB, Burton and colleagues analysed MRI scans of twenty three patients with DLB and corresponding post-mortem brain tissue (Burton et al. 2012). Reduced amygdala volume on MRI correlates with Lewy body pathology, while hippocampal and entorhinal cortex volumes do not show a direct relationship with Lewy body or Alzheimer’s pathology suggesting other pathological factors may be influencing atrophy in these brain regions.

In addition, Kantarci and colleagues discovered antemortem hippocampal and amygdala volumes increased from low to intermediate to high likelihood of DLB, however smaller hippocampal and amygdala loads were associated with higher neurofibrillary tangle stage suggesting concomitant AD related pathology could impact atrophy rates in DLB cases (Kantarci et al. 2012). Further to this, Nedelska and colleagues investigated the pattern and magnitude of the atrophy rates from two serial MRIs in autopsy-confirmed DLB, mixed DLB/AD, and elderly non-demented controls. In DLB patients, the atrophy rates correlated with Braak neurofibrillary tangle stage suggesting global and regional atrophy rates are associated with AD-type pathology in DLB, and can be used as biomarkers of AD progression in patients with Lewy body pathology (Nedelska et al. 2014).

To determine the structural MRI and neuropathological correlates of rapid eye movement (REM) sleep behaviour disorder (RBD), a core diagnostic feature in DLB patients, Murray and colleagues used a cohort of 75 prospectively followed patients that neuropathologically confirmed brainstem, limbic or diffuse LBD and had undergone 1.5 Tesla MRI scan during life. They found that patients that had RBD had lower Aβ and phosphorylated tau pathology in the hippocampus and amygdala whilst in patients without RBD greater atrophy in the temporoparietal, hippocampus and amygdala was observed (Murray et al. 2013). The authors conclude RBD status and medial temporal lobe atrophy on MRI may have implications for identifying patients who may benefit from treatments targeting disease-specific proteins.

#### Fluorodeoxyglucose Positron Emission Tomography

The cingulate island sign (CIS) is a supportive imaging biomarker that discriminates DLB from AD and refers to the preservation of metabolism in the posterior cingulate cortex. The neuropathological basis of CIS is still currently unknown, however a study by Graff-Radford determined patients with a higher CIS ratio had lower neurofibrillary tau tangle scores, but there was no difference in the CIS ratio between transitional and diffuse LBD (Graff-Radford et al. 2014, 2020). Further to this Patterson explored other neurogenerative pathologies in an autopsy cohort of DLB, AD, and control cases that had underwent ^99m^Tc-hexamethylpropyleneamine oxime (HMPAO) single-photon emission computed tomography imaging during life. They found no relationship between CIS ratios and hyperphosphorylated tau, Aβ, or α-synuclein in the posterior cingulate cortex (Patterson et al. 2019). However, the anterior cingulate cortex displayed a lower HMPAO uptake, and higher α-synuclein in DLB which the authors suggest could signify the relative preservation of perfusion in the posterior cingulate cortex compared to the anterior cortex. This pathological finding supports a hypothesis from Kameyama and Iizuka that hypoperfusion/hypometabolism in the posterior cingulate cortex as indicated by the CIS ratio being attributable to diaschisis resulting from damage in the nucleus basalis of Meynert and not local changes to neurodegenerative pathologies (Kameyama and Iizuka 2020).

## Novel biomarkers

### Transcranial Sonography

Transcranial sonography (TCS) is an ultrasound technique used to visualise deep brain structures in particular the SN. Displaying tissue hyperechogenicity in the brain of PD patients it is thought to reflect an increased burden of iron (Becker et al. 1995; Zhang et al. 2024). It’s relevance to nigral degeneration has been investigated in a cohort of 50 patients with PD, Weise and colleagues demonstrated extension of echogenic SN area inversely correlated with striatal activity of presynaptic dopamine reuptake transporter (Weise et al. 2009).

Walter and colleagues demonstrated that SN hyperechogenicity is present in approximately 80% of patients with DLB (Walter et al. 2006).

Although there are limited data directly correlating TCS with neuropathological changes in LBD, Berg and colleagues performed TCS on 20 patients without extrapyramidal and subsequent heavy metal and histological examination on post-mortem brain tissue. Within the post-mortem tissue echogenicity of the SN correlated with its iron content (Berg et al. 2002).

### Quantitative susceptibility mapping

Quantitative susceptibility mapping (QSM), an MRI-based technique has also been identified as a method to assess abnormal iron burden in α -synucleinopathies. In a multimodal study combining QSM and TCS, serum iron markers, and clinical assessment in PD cases, substantia nigra susceptibility correlated with echogenic area and echo intensity. Mean bilateral susceptibility also correlated with transferrin saturation and showed a trend with serum iron (Luyken et al. 2025).

Capogna and colleagues used QSM for in vivo assessment of brain iron deposition in DLB patients and older adults. DLB patients showed higher magnetic susceptibility values than older adults in the bilateral substantia nigra, globus pallidus, thalamus, brainstem, and hippocampus, as well as in posterior cortical regions (Capogna et al. 2026).

QSM has been validated in LBD in a post-mortem study. Diaz-Galvan and colleagues demonstrated autopsy confirmed LBD cases had significantly higher QSM values in the substantia nigra compared with the non-LBD group. Subregional analyses revealed that this difference was specific to the substantia nigra pars compacta, with no significant variation observed in the pars reticulata. Moreover, QSM measurements of the substantia nigra pars compacta discriminated between LBD and non-LBD cases (Diaz-Galvan et al. 2025). To date validation, post-mortem iron assessment in DLB is still lacking.

The biomarkers discussed above all measure neuronal death or dysfunction secondary to α-synuclein pathology. More recently, imaging markers are emerging to measure α-synuclein pathology directly.

### α-synuclein PET tracers

As the hallmark pathology that identifies people that have DLB, radioligands that can selectively target α-synuclein deposits would provide critical tools to aid accurate diagnostic tools. However, PET tracers for α-synuclein have been elusive, partly due to the sparse burden of deposits in the brain. In addition, they have to be highly specific and not have an affinity for either Aβ or tau, which are frequently observed in DLB. There are a number of radioligands in development including F0502B, C05-05, and AC1-12589 (Smith et al. 2023; Xiang et al. 2023; Endo et al. 2024).

Autoradiography has demonstrated that ^18^F-F0502B can selectively bind with Lewy bodies in the striatum and SN region in PD and barely detects amyloid plaques or neurofibrillary tangles in AD (Xiang et al. 2023). To further verify the ^18^F‐F0502B diagnostic potential, Xiang and colleagues generated two PD non‐human primate models by injecting α‐synuclein pre-formed fibrils or adeno‐associated virus α‐synuclein A53T into the striatum. PET imaging demonstrated that [^18^F]‐F0502B specifically recognizes α‐synuclein aggregates in the brains of monkeys. These data taken together suggest [^18^F]‐F0502B is relevant for brain regions and inclusions associated with the LBDs.

Endo and colleagues describe a C05 series compound, (*E*)-1-fluoro-3-((2-(4-(6-(methylamino)pyridine-3-yl)but-1-en-3-yn-1-yl)benzo[*d*]thiazol-6-yl)oxy)propan-2-ol (named C05-05) that has potential binding properties as an in vitro agent for α-synuclein PET (Endo et al. 2024). They assessed binding of ^18^F-C05-05 to human α-synuclein pathologies in basal ganglia from PDD and multiple system atrophy (MSA) and amygdala from a DLB case in autoradiography experiments. Whilst 2/3 MSA cases demonstrated positive binding with glial cytoplasmic inclusions, the DLB case displayed specific binding with α-synuclein pathology within the amygdala. PET detectable ^18^F-C05-05 signals were also detectable in patients at a greater intensity compared to controls.

Smith and colleagues demonstrated elevated [^18^F]ACI-12,589 in a monogenic PD case carrying a G51D mutation in the *SNCA* gene, however this intensity was not observed in idiopathic PD and DLB cases, most likely due to more dense and widespread α-synuclein pathology in the familial case compared to idiopathic PD cases (Smith et al. 2023). The development of α-synuclein PET tracers is still in its infancy and more progress needs to be made before it can be useful in the clinic.

### Imaging of co-pathologies

It is well known that the predominant pathological protein deposition in DLB consists of α-synuclein. However, there is considerable heterogeneity, particularly regarding the burden of AD related neuropathology (tau and amyloid β) (Irwin et al. 2017; Coughlin et al. 2019; Walker et al. 2019; McAleese et al. 2021). One neuropathological study demonstrated 100% of DLB cases had at least Braak neurofibrillary tangle stage I, and 95% of cases exhibiting at least Thal phase 1 (Walker et al. 2019). In addition, up to 50% of DLB cases have high AD neuropathologic change (Tiraboschi et al. 2015; Walker et al. 2019), driving progression of pathology and accelerating cognitive decline (Irwin et al. 2017; Irwin and Hurtig 2018; Walker et al. 2019). This pathologic overlap complicates the differential diagnosis of DLB (Thomas et al. 2018).

#### Amyloid PET

Amyloid PET is a powerful tool that provides direct visualization of fibrillar Aβ in AD (Rabinovici et al. 2025). The development of Aβ PET radiopharmaceuticals began with the ^11^C-Pittsburgh compound B (PiB), which was followed by the more widely used ^18^F-labeled agents such as florbetapir, florbetaben, flutemetamol (Skyles et al. 2026). Post-mortem studies have demonstrated that Aβ PET imaging correlates well with Aβ deposition in the form of neuritic and diffuse plaques, and amyloid angiopathy (Ikonomovic et al. 2008; Sojkova et al. 2011; Clark et al. 2012). As a co-pathology marker, Aβ PET is probably the most widely studied ligand in DLB, and it seems essential that Aβ can be identified in vivo considering the putative synergistic relationship that exists between Aβ and α-synuclein (Lashley et al. 2008; Swirski et al. 2014) and the increased cognitive impairment associated with the cumulative burden of Aβ, α‐synuclein, and tau in LBD (Howlett et al. 2015). Interestingly, in a clinical-imaging study Donaghy and colleagues did not find any differences in cognitive or neuropsychiatric domains in DLB patients with Aβ positive and negative PET scans (Donaghy et al. 2018). However in a longitudinal follow up study, Aβ deposition on visual rating was associated with greater decline in Mini-Mental State Examination and daily function over 1 year in patients with DLB, providing further evidence for a link between Aβ deposition and clinical progression in DLB (Donaghy et al. 2020).

Using post-mortem confirmed DLB cases who underwent antemortem PiB-PET Kantarchi and colleagues were able to show lower PiB uptake accurately distinguishes cases with LBD from cases with AD or mixed pathology, correlating with the Thal Aβ phase (Kantarci et al. 2020). This demonstrates amyloid PET can be a clinically relevant biomarker of Aβ pathology in DLB. This will have implications for anti-amyloid therapy should it be approved for use in DLB. In the Clarity AD study, (a phase 3 clinical trial to assess the safety and efficacy of Lecanamab (a humanized monoclonal antibody that binds with high affinity to soluble Aβ protofibrils) secondary endpoints demonstrated a decrease in amyloid burden on PET in patients on the active drug compared to placebo (van Dyck et al. 2022).

#### Tau PET

Post-mortem data and cerebrospinal fluid studies demonstrated the influence of comorbid tau on clinical heterogeneity, brain imaging, and survival rates (Nelson et al. 2012; Howlett et al. 2015; Irwin et al. 2017; Coughlin et al. 2019). Therefore, it is critical that we can identify tau accumulation in patients with DLB so patients can be accurately enrolled into clinical trials and disease modifying therapies can be given when available.

Tau PET (particularly 18 F-flortaucipir) is currently approved by the Food and Drug Administration for patients undergoing evaluation for AD (Jie et al. 2021) and is part of a biomarker panel to assess endpoints for tau antibody therapies (Racine et al. 2021; Shulman et al. 2023). It demonstrates high affinity for AD related aggregates (Malpetti, La Joie et al. 2022) and is able to discriminate DLB from AD (Kantarci et al. 2017; Ossenkoppele et al. 2018). However, studies demonstrate little to no elevation of tau PET in DLB compared to normally aged controls (Mak et al. 2021) and it is not specific for less mature tau aggregates in tauopathies (Lowe et al. 2016). Tau pathology in the form of neurofibrillary tangles have a dynamic lifespan of maturity existing in a spectrum from early to mature aggregates. It seems reasonable that tau radioligands also capture different stages of tau aggregate maturity, and if ligands have an affinity for specific conformational states of tau i.e. mature aggregates, immature aggregates may not be detected by tau PET. To ensure appropriate patients are being enrolled into clinical trials, we need to be confident tools that are used to determine trial inclusion suitability are appropriate and effective at detecting the accurate pathological load within each patient. To date no robust post-mortem/neuroimaging correlation study has been conducted in a DLB cohort with varying burdens of tau pathology.

### Markers of neuroinflammation

#### 18 kDa translocator protein PET

Neuroinflammation can play a key role in the pathogenesis of AD (Heneka et al. 2015, 2025) and in a neuropathological study in DLB, microglial activation was largely associated with AD co-pathology (van Wetering, Geut et al. 2025). Post-mortem studies in established DLB have demonstrated that microglial activation is not a prominent feature of end-stage DLB (Amin et al. 2020), however in mild cognitive impairment with Lewy bodies (MCI-LB) significantly higher levels of interleukins IL-10, IL-1β, IL-4 and IL-2 was increased compared to controls with no difference between controls and established DLB cases in plasma samples (King et al. 2018). These findings raise the possibility that inflammatory processes in the central nervous system may be an important pathophysiological early driver in the development of DLB.

The 18 kDa Translocator Protein (TSPO), also originally called peripheral benzodiazeine binding receptors (PBR) is a mitochondrial protein expressed by microglia and is used as a radioligand in neurodegenerative diseases as a marker of inflammation. In a neuroimaging study with complementary peripheral blood cytokine analysis Surendranathan and colleagues used ^11^C-PK11195 (a first generation TSPO radioligand) and found microglial activation was elevated in dementia with Lewy bodies subjects with mild disease when compared to those with moderate/severe impairment (Surendranathan et al. 2018), which lends support to the hypothesis that DLB is associated with early microglial activation in key areas affected by DLB pathology. In a neuropathological study to test the hypothesis that ante-mortem TSPO PET imaging correlates with regional and individual post-mortem differences in microglia in the tauopathy progressive supranuclear palsy (PSP), Wijesinghe and colleagues found a significant positive correlation between regional ^11^C-PK11195 binding potential ante-mortem and the burden of post-mortem CD68 + phagocytic microglia, as well as microglial TSPO levels in PSP patients. This suggests TSPO is a biomarker for microglia-mediated neuroinflammation in tauopathies (Wijesinghe et al. 2025). However to date, no post-mortem studies have investigated microglial activity in MCI-LB/mild DLB.

#### Emerging targets of interest

The overexpression of TSPO is well established in identifying microglia, however it does not capture the full spectrum of inflammatory processes in the brain such as changes in different microglial phenotypes (resting, pro-inflammatory, anti-inflammatory) or changes in astrocytes. Therefore, our understanding of inflammatory dynamics will be increased with alternative targets for PET imaging (see review (Chauveau et al. 2025). Emerging microglial PET tracers include P2Y12R, P2 × 7R, CSF1R, and COX, whilst MAO-B and I2BS are targets for astrocytes. Although yet to be trialled in DLB patients, autoradiography studies suggest [(11)C]-L-deprenyl, the PET radioligand of MAO-B, is negatively correlated with Braak neurofibrillary tangle stage, which suggests it could be a promising marker of astrocytes in DLB where inflammation has been hypothesised and an early prodromal event in the disease process (King et al. 2018). Further post-mortem/neuroimaging studies are required to determine the neuropathological correlates of emerging PET ligands.

There are suggested links between iron and inflammation in PD (Song et al. 2018; Riederer et al. 2023) and AD (Hosseini et al. 2026). Multimodal imaging studies with post-mortem validation are needed to investigate the link between iron deposition and neuroinflammation.

## Limitations

To better determine the neuropathological correlates of neuroimaging changes in DLB the research field requires cases that have both ante-mortem imaging and full neuropathological assessment of the brain following post-mortem donation.

All of the studies outlined in this review are limited by the number of cases included. Another challenge is the time after the last brain scan to brain donation as patients can often live years following their final scan and lead to subsequent changes in neuropathology. One important patient demographic that is not included in many scientific studies is the race and ethnicities of the participants. Post-mortem studies have reported that mixed pathologies are more likely in Black and Hispanic participants compared to White counterparts (Barnes et al. 2015; Filshtein et al. 2019), therefore it is vital when recruiting for neuroimaging studies and brain donation programmes that we increase diversity of participants to represent a worldwide population that will cover the heterogeneity that is commonly seen in DLB.

## Future directions

Taken together, recent advances in molecular imaging are reshaping how LBD is conceptualised, studied, and ultimately diagnosed (Table 1). Future progress will depend on tightly integrated multimodal imaging and linking protein aggregation to network dysfunction and cognitive decline. Quantitative neuropathological validation studies of emerging imaging targets, particularly those targeting α-synuclein burden, synaptic dysfunction, and co-pathology, will aid the understanding of the heterogeneity in the full LBD spectrum. Looking ahead, applying these tools to prodromal and pre-dementia stages will be essential for redefining disease onset, refining diagnostic criteria, and enabling disease-modifying trials.

**Table 1:** Summary of neuroimaging modalities in research and clinical use in DLB

Imaging Modality	Target Pathology	Neuropathological Validation	Diagnostic Utility	Current Limitations
Striatal dopaminergic imaging	Nigrostriatal dopaminergic neurones	Strong correlation with nigrostriatal degeneration and loss of dopaminergic neurons	-Indicative biomarker for DLB. -Differentiates DLB from AD and non-degenerative parkinsonism	-Limited for disease staging - False negatives in early disease
(^123^I-MIBG) myocardial scintigraphy	Cardiac sympathetic innervation	Strongly correlates with postganglionic sympathetic denervation	- Indicative biomarker in DLB criteria - Distinguishes DLB from AD	- Affected by co-morbidities i.e. cardiac disease, diabetes, medications - Not widely available
Structural MRI	Brain atrophy patterns	Moderate correlation with neuronal loss, though DLB shows relatively preserved medial temporal lobe	-Supportive marker, helps exclude other causes - Relative preservation of hippocampus supports DLB vs. AD	- Low sensitivity and specificity for DLB - Often normal early in disease
FDG-PET	Cerebral glucose metabolism	Reflects synaptic dysfunction and neuronal loss; occipital hypometabolism aligns with Lewy body pathology distribution	-Supportive marker (occipital hypometabolism and cingulate island sign) - Helps differentiate DLB from AD	- Not specific (overlap with other dementias) - Limited availability
α-synuclein PET	Aggregated α-synuclein (Lewy bodies/neurites)	Theoretically direct measure of core DLB pathology, but robust in vivo clinicopathological validation is still lacking	- Novel, experimental biomarker - Could enable early diagnosis, disease tracking, and therapy monitoring	- No widely validated or clinically available tracer yet - Challenges: low target density, intracellular location, and off-target binding - Currently only in a research setting
Amyloid PET	β-amyloid deposition	Correlates with amyloid plaque burden, a common co-pathology in DLB	-Helps identify mixed DLB + AD pathology	Limited availability
Tau PET	Tau (neurofibrillary tangles)	Correlates with tau pathology often a co-pathology in DLB	- Helps assess co-existing AD pathology - May aid differential diagnosis	- Limited relevance to core DLB pathology
TSPO PET (e.g. ¹¹C-PK11195, ¹⁸F-DPA-714)	Microglial activation (neuroinflammation)	Correlates with activated microglia largely in PSP post-mortem studies, studies in DLB are lacking	- Potential early marker of neuroinflammation - Insight into disease mechanisms and progression	- Non-specific as inflammation present in many neurodegenerative diseases - Currently only in a research setting
Astrocyte PET (emerging, e.g. MAO-B tracers)	Astrocyte reactivity	Associated with reactive astrocytosis observed in neurodegeneration	Research tool for glial contribution to DLB	- Very early-stage - Minimal DLB-specific validation - Currently only in a research setting
Transcranial sonography	Echogenicity of substantia nigra	Associated with iron deposition and nigral degeneration	- May support parkinsonian syndromes	- Limited evidence in autopsy-confirmed DLB - Currently only in a research setting
Quantitative Susceptibility Mapping	Magnetic susceptibility, primarily iron deposition)	Increasing evidence linking iron accumulation to α-synuclein aggregation, oxidative stress, and neurodegeneration, though direct clinicopathological validation in DLB remains limited compared to PD	- Potential to detect nigral and striatal iron changes - May aid differentiation of parkinsonian syndromes - Potential biomarker for disease mechanisms	- Limited validation in autopsy-confirmed DLB cohorts - Overlap with other neurodegenerative diseases - Currently only in a research setting

DaT; dopamine transporter, SPECT; Single Photon Emission Computed Tomography, DLB, dementia with Lewy bodies, AD; Alzheimer’s disease, PD; Parkinson’s disease, ^123^I-MIBG; ^123^I-metaiodobenzylguanidine, MRI; magnetic resonance imaging, FDG-PET; Fluorodeoxyglucose Positron Emission Tomography, TSPO; 18 kDa Translocator Protein, MAO-B; Monoamine oxidase B

## Data Availability

No datasets were generated or analysed during the current study.
